# Characterization of human T cell receptor repertoire data in eight thymus samples and four related blood samples

**DOI:** 10.1016/j.dib.2021.106751

**Published:** 2021-01-20

**Authors:** Nelli Heikkilä, Iivari Kleino, Reetta Vanhanen, Dawit A. Yohannes, Ilkka P. Mattila, Jari Saramäki, T. Petteri Arstila

**Affiliations:** a Research Programs Unit, Translational Immunology, University of Helsinki, Haartmaninkatu 3, 00290 Helsinki, Finland; b Medicum, Department of Bacteriology and Immunology, University of Helsinki, Haartmaninkatu 3, 00290 Helsinki, Finland; c Department of Medical and Clinical Genetics, University of Helsinki, Haartmaninkatu 8, 00290 Helsinki, Finland; d Department of Pediatric Cardiac and Transplantation Surgery, Hospital for Children and Adolescents, Helsinki University Central Hospital, Stenbäckinkatu 9, 00290 Helsinki, Finland; e Department of Computer Science, Aalto University, Konemiehentie 2, 02150 Espoo, Finland

**Keywords:** TCR repertoire, CDR3, Public immune responses, thymus

## Abstract

T cell receptor (TCR) is a heterodimer consisting of TCRα and TCRβ chains that are generated by somatic recombination of multiple gene segments. Nascent TCR repertoire undergoes thymic selections where non-functional and potentially autoreactive receptors are removed. During the last years, the development of high-throughput sequencing technology has allowed a large scale assessment of TCR repertoire and multiple analysis tools are now also available.

In our recent manuscript, *Human thymic T cell repertoire is imprinted with strong convergence to shared sequences*[1], we show highly overlapping thymic TCR repertoires in unrelated individuals. In the current Data in Brief article, we provide a more detailed characterization of the basic features of these thymic and related peripheral blood TCR repertoires. The thymus samples were collected from eight infants undergoing corrective cardiac surgery, two of whom were monozygous twins [2]. In parallel with the surgery, a small aliquot of peripheral blood was drawn from four of the donors. Genomic DNA was extracted from mechanically released thymocytes and circulating leukocytes. The sequencing of TCRα and TCRβ repertoires was performed at ImmunoSEQ platform (Adaptive Biotechnologies). The obtained repertoire data were analysed applying relevant features from immunoSEQ® 3.0 Analyzer (Adaptive Biotechnologies) and a freely available VDJTools software package for programming language R [3].

The current data analysis displays the basic features of the sequenced repertoires including observed TCR diversity, various descriptive TCR diversity measures, and V and J gene usage. In addition, multiple methods to calculate repertoire overlap between two individuals are applied. The raw sequence data provide a large database of reference TCRs in healthy individuals at an early developmental stage. The data can be exploited to improve existing computational models on TCR repertoire behaviour as well as in the generation of new models.

## Specifications Table

**Table:** 

Subject	Immunology
Specific subject area	T cell antigen receptor (TCR) alpha chain and beta chain diversity and characteristics in thymus and in peripheral blood
Type of data	Table: Sample description by immunoSEQ and VDJTools softwares (Table 1), repertoire diversity metrics (Table 2), resampled repertoire diversity metrics (Table 3), repertoire overlap measures (Table 4). Graph: V gene usage heatmap (Figure 1), J gene usage heatmap (Figure 2), rarefaction plots (Figure 3), clustering of overlap analyses (Figure 4).
How data were acquired	TCRAD and TCRB sequencing was performed at ImmunoSEQ platform (Adaptive Biotechnologies). TCR analysis was performed using immunoSEQ® 3.0 Analyzer (Adaptive Biotechnologies) and VDJTools software [3].
Data format	Raw Analysed
Parameters for data collection	Thymus samples were obtained from eight immunologically healthy infants undergoing open cardiac surgery for congenital heart defects. A small aliquot of blood (0.5–1 mL) was drawn from four subjects during the operation. The study was approved by the Pediatric Ethical Committee of the Helsinki University Hospital (HUS/747/2019) and a written informed consent was obtained from the parents.
Description of data collection	Thymocytes were extracted mechanically from tissue resects. Blood samples were treated with ACK lysis buffer (Thermo Fisher Scientific) to remove erythrocytes. DNA was extracted from 10–30 million thymocytes and from all available PBMCs. TCRAD and TCRB sequencing was performed as previously described [4] from a standardized quantity of genomic DNA using ImmunoSEQ assay (Adaptive Biotechnologies), which exploits a multiplex PCR system spanning the V(D)J region at a length that is sufficient to identify V and J genes and cover unique CDR3 regions.
Data source location	Institution: University of Helsinki City/Town/Region: Helsinki Country: Finland
Data accessibility	Repository name: The European Nucleotide Archive (ENA) at EMBL-EBI Data identification number: PRJEB41936 Direct URL to data: https://www.ebi.ac.uk/ena/browser/view/PRJEB41936
Related research article	Heikkilä Nelli, Vanhanen Reetta, Yohannes Dawit A., Kleino Iivari, Mattila Ilkka P., Saramäki Jari, Arstila T. Petteri Human thymic T cell repertoire is imprinted with strong convergence to shared sequences Molecular Immunology, Volume 127, November 2020, Pages 112–123 doi.org/10.1016/j.molimm.2020.09.003

## Value of the Data

• These data consist of a unique collection of over 62 million T cell receptor (TCR) sequences obtained directly from human thymus. It is a large scale resource of human TCRα and TCRβ repertoires at an early developmental stage before clonal selections by peripheral antigens and devoid of medical or immunological interventions.
• The data are useful for those who wish to compare TCR repertoires from healthy thymus and from individuals affected by immunological diseases or other medical conditions. The large scale thymic repertoire data can also benefit computational experiments which have been typically limited to peripheral blood TCR data.
• These data can be directly exploited to improve existing computational models on TCR repertoire generation as well as in the generation of new models. These data can also guide design of human TCR sequencing experiments and serve as a reference database for new experiments.

## 1 Data Description

**Fig. 1:**
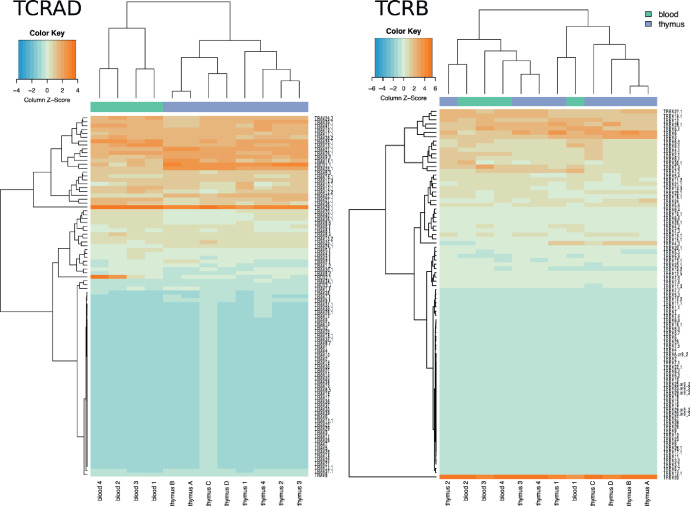
The V gene usage in TCRAD and TCRB repertoires. Z-scores indicate the relative frequency of each segment. Dendrograms show clustering of the samples and the gene segments.

**Fig. 2:**
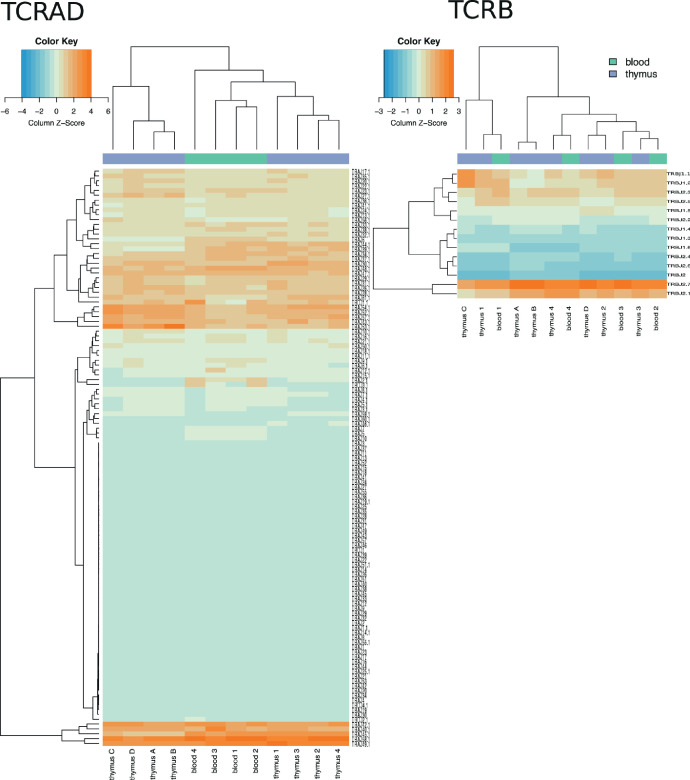
The J gene usage in TCRAD and TCRB repertoires. Z-scores indicate the relative frequency of each segment. Dendrograms show clustering of the samples and the gene segments.

All TCRAD and TCRB sequences obtained from eight thymus (donors A-D and donors 1–4) and four related blood samples (donors 1–4) have been deposited in the European Nucleotide Archive (ENA) at EMBL-EBI under accession number PRJEB41936 (https://www.ebi.ac.uk/ena/browser/view/PRJEB41936). In addition, the sequences are available at immuneACCESS® repository in the form of immunoSEQ^TM^ output format and can also be downloaded as raw FASTA files (https://clients.adaptivebiotech.com/pub/heikkila-2020-mi). On average, we obtained 4.1 million unique TCRα and 810 000 unique TCRβ clonotypes from each thymus. From blood samples we obtained on average 150 000 and 84 000 unique TCRα and TCRβ sequences, respectively. An overview of sequence diversities, total counts and sequence productivity (in-frame vs. non-coding sequences) was generated both by immunoSEQ^TM^ and VDJTools softwares and is displayed together with donor details in Table 1. Two of the donors (A and B) were monozygous twins and the influence of genetics in the repertoire has been analysed previously [2]. The V and J gene usage has been shown to be biased in the peripheral blood but also already in the thymus [5], [6], [7]. The gene segment usage in the current samples is also biased (Figs. 1 & 2).

**Table 1:** Description of the sequenced samples.

TCRAD											
Sample id	Age (days)	Sex	Tissue	immunoSEQ^TM^: count	VDJTools: count	immunoSEQ^TM^: diversity	VDJTools: diversity	immunoSEQ^TM^: non-coding diversity	VDJTools: non-coding diversity	immunoSEQ^TM^: non-coding frequency	VDJTools: non-coding frequency
thymus A	243	M	thymus	11 838 086	11 838 086	6 907 422	6 763 870	4 719 902	2 090 241	68.33%	31.10%
thymus B	244	M	thymus	12 849 473	12 849 473	7 578 104	7 419 245	5 179 754	2 307 719	68.35%	31.22%
thymus C	225	F	thymus	8 359 283	8 359 283	5 347 824	5 259 057	3 663 398	1 752 208	68.50%	33.98%
thymus D	126	M	thymus	11 063 464	11 063 464	6 743 495	6 610 182	4 617 533	2 007 905	68.47%	30.29%
thymus 1	7	M	thymus	3 179 774	3 179 774	2 089 557	1 984 292	1 447 726	601 486	69.28%	30.01%
thymus 2	52	M	thymus	1 747 487	1 747 487	1 262 845	1 198 677	883 536	385 749	69.96%	32.05%
thymus 3	107	M	thymus	2 158 043	2 158 043	1 289 728	1 230 436	902 934	398 227	70.01%	32.24%
thymus 4	156	F	thymus	1 848 851	1 848 851	1 419 013	1 345 927	997 764	441 964	70.31%	32.71%
blood 1	7	M	blood	154 682	154 682	138 159	130 307	86 201	34 147	62.39%	26.02%
blood 2	52	M	blood	123 523	123 523	109 171	103 142	65 096	26 413	59.63%	25.12%
blood 3	107	M	blood	245 126	245 126	180 100	170 333	110 571	45 852	61.39%	24.94%
blood 4	156	F	blood	199 326	199 326	167 266	157 728	104 846	45 404	62.68%	27.89%

TCRB											
Sample id	Age (days)	Sex	Tissue	immunoSEQ^TM^: count	VDJTools: count	immunoSEQ^TM^: diversity	VDJTools: diversity	immunoSEQ^TM^: non-coding diversity	VDJTools: non-coding diversity	immunoSEQ^TM^: non-coding frequency	VDJTools: non-coding frequency

thymus A	243	M	thymus	1 647 656	1 647 656	1 254 760	1 245 029	288 199	108 933	22.97%	8.46%
thymus B	244	M	thymus	1 783 878	1 783 878	1 540 161	1 526 694	363 551	138 386	23.60%	8.89%
thymus C	225	F	thymus	1 850 299	1 850 299	1 568 528	1 551 603	248 898	93 724	15.87%	5.89%
thymus D	126	M	thymus	1 726 796	1 726 796	1 462 150	1 449 881	279 672	106 019	19.13%	7.15%
thymus 1	7	M	thymus	237 063	237 063	223 725	222 925	53 389	19 585	23.86%	8.76%
thymus 2	52	M	thymus	182 356	182 356	173 368	172 746	35 779	14 443	20.64%	8.28%
thymus 3	107	M	thymus	142 903	142 903	138 544	137 920	31 385	12 183	22.65%	8.75%
thymus 4	156	F	thymus	128 228	128 228	122 195	121 483	25 475	10 129	20.85%	8.24%
blood 1	7	M	blood	82 418	82 418	77 868	77 281	21 203	7 462	27.23%	9.51%
blood 2	52	M	blood	73 945	73 945	69 875	69 404	17 566	6 783	25.14%	9.81%
blood 3	107	M	blood	134 110	134 110	104 236	103 551	26 162	9 831	25.10%	8.73%
blood 4	156	F	blood	88 901	88 901	82 550	81 935	20 852	8 151	25.26%	10.19%

**Fig. 3:**
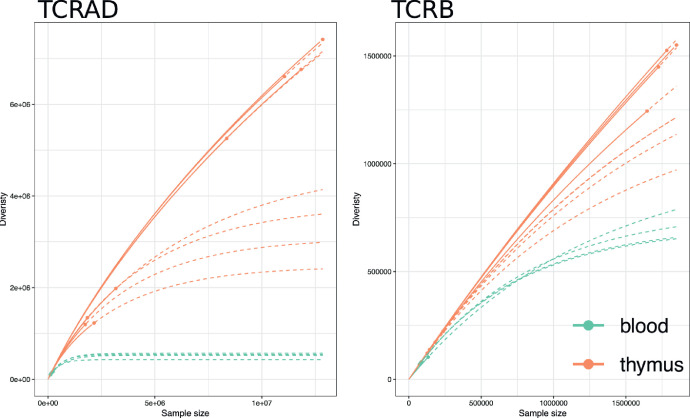
The rarefaction curves of TCRAD and TCRB diversities for each sample. The thymic samples are marked in red and peripheral samples in blue. The dots indicate observed diversity and counts, solid lines interpolated and dashed lines extrapolated values.

The TCR diversity has been previously assessed both in the peripheral blood and in the thymus and multiple diversity metrics are available [4,[8], [9], [10]. The diversity estimates for the current samples were calculated using VDJTools software with default settings. To estimate the lower bound of total species richness, VDJTools provide unmodified Chao1, extrapolated Chao (chaoE) and Efron-Thisted estimates while the repertoire diversity is depicted with Shannon's index and inverse Simpson's index (Table 2). The species richness and repertoire diversity indexes are also calculated for datasets down-sampled to the size of the smallest dataset to facilitate the comparison of samples with different sequencing depths (Table 3). Furthermore, a rarefaction curve based on the relationship between the sample diversity and the sample size was plotted for TCRα and TCRβ with extrapolation to the size of the largest sample (Fig. 3).

**Fig. 4:**
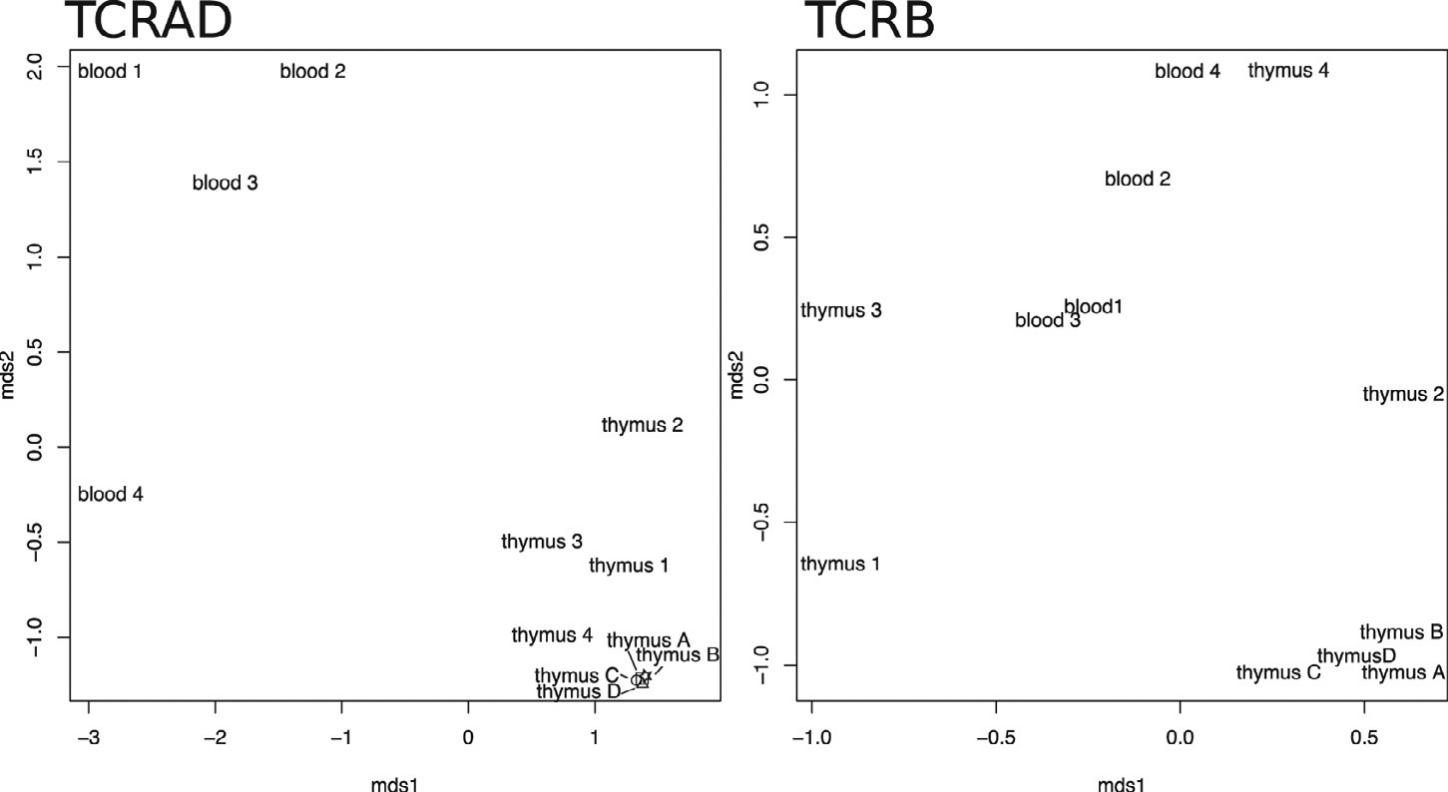
Clustering of the samples on multi-dimensional scaling according to pairwise repertoire overlap comparison with Jaccard index.

Despite the high potential diversity of TCR repertoires, a surprisingly high fraction of the repertoire is shared between individuals [1]. Here, we calculated various overlap measures with VDJTools: Pearson correlation, relative overlap measure [rationale explained in 11], Jaccard index and Morisita-Horn index (Table 4). The calculations were performed on the entire repertoire and exact matching of V gene, J gene and the CDR3 region was required. The clustering of different samples with multidimensional scaling is depicted for Jaccard index (Fig. 4).

## 2 Experimental Design, Materials and Methods

Thymus samples were obtained from eight immunologically healthy infants undergoing corrective cardiac surgery for congenital heart defects. The study was approved by the Pediatric Ethical Committee of the Helsinki University Hospital (HUS/747/2019). A written informed consent was obtained from the parents. Thymocytes were extracted mechanically from tissue resects and stored as pellets of 10–30 million thymocytes in −70 °C. From four donors a small aliquot of 0.5–1 mL peripheral blood was drawn during the surgery. To remove erythrocytes, the blood samples were treated with ACK lysis buffer (Thermo Fisher Scientific, USA) according to manufacturer's orders and the obtained leukocytes were stored as pellets in −70 °C. Genomic DNA was extracted from frozen pellets with QIASymphony^TM^ (Qiagen, Germany) according to manufacturer's orders. TCRAD and TCRB regions were sequenced from a standardized quantity of quality-controlled genomic DNA using ImmunoSEQ^TM^ assay (Adaptive Biotechnologies). The assay uses a multiplex PCR system spanning the TCRAD VJ and TCRB VDJ regions at a length that is sufficient to cover unique CDR3 regions and to identify V and J genes. Amplicon sequencing was performed on Illumina platform. TCRAD and TCRB definitions were based on IMGT database (www.imgt.org). Primer bias and sequencing errors were corrected as previously described [4].

For each sequenced sample the ImmunoSEQ^TM^ assay outputs a file of unique nucleotide sequences covering V and J genes and the CDR3 region, the count and frequency of each sequence, the CDR3 region length, and whether the sequence is in-frame, out-of-frame or contains a premature STOP codon. For in-frame and ‘has stop’ sequences the nucleotide sequence is converted to CDR3 amino acid sequence and * symbol indicates the STOP codon. In addition, the V gene, D gene and J gene names, the number of non-templated nucleotide insertions and the locations of insertions in V and J gene segments are provided. The raw FASTA files are also available but not directly used in the present analysis.

In the current article we applied TCR analysis tools form two platforms: immunoSEQ^TM^ ANALYZER 3.0 run on Adaptive Biotech website (adaptivebiotech.com/products-services/immunoseq/immunoseq-analyzer/) and a java based non-commercial software package VDJTools [3]. From immunoSEQ^TM^ we adapted “Sample Overview” to calculate the sample diversity and counts. VDJTools readily accepts the basic immunoSEQ^TM^ output format and converts it to a VDJTools output file. From VDJTools we used “CalcBasicStats” command to calculate the sample diversity and counts, “CalcSegmentUsage” command to produce V and J gene usage heatmaps, “CalcDiversityStats” and “RarefactionPlot” commands with default settings to calculate and visualise diversity estimations, and finally “CalcPairwiseDistances” command to calculate the sequence overlap between two samples. For sequence overlap we selected the setting “strict”, which requires matching CDR3 nucleotide regions as well as matching V genes and J genes. For visualisation of Jaccard index overlap values we used “ClusterSamples” tool that provides a multi-dimensional scaling plot created with isoMDS() function of MASS package for R.

**Table 2:** Diversity estimates.

TCRAD										
Sample id	Age (days)	Sex	Tissue	Observed counts	Observed diversity	Chao1 (mean±std)	Extrapolated ChaoE (mean±std)	Efron-Thisted (mean±std)	Shannon's index (mean)	Inversed Simpson's index (mean)
thymus A	243	M	thymus	11 838 086	6 763 870	16 826 997 ± 13 658	7 160 320 ± 2 017	46 864 937 ± 2 469 178	4 617 696	1 633 144
thymus B	244	M	thymus	12 849 473	7 419 245	18 828 064 ± 14 772	7 419 245 ± 2 120	41 786 084 ± 2 534 757	5 058 759	1 796 531
thymus C	225	F	thymus	8 359 283	5 259 057	14 602 322 ± 14 425	7 124 951 ± 2 068	45 140 688 ± 3 293 359	3 861 431	1 603 852
thymus D	126	M	thymus	11 063 464	6 610 182	16 698 624 ± 13 750	7 342 590 ± 2 021	23 898 564 ± 1 279 838	4 677 030	1 764 774
thymus 1	7	M	thymus	3 179 774	1 984 292	4 754 478 ± 6 900	4 141 693 ± 3 605	11 705 484 ± 692 435	1 588 328	1 011 926
thymus 2	52	M	thymus	1 747 487	1 198 677	3 089 405 ± 5 992	2 989 346 ± 4 808	7 000 872 ± 416 869	1 017 426	771 547
thymus 3	107	M	thymus	2 158 043	1 230 436	2 468 027 ± 3 953	2 410 929 ± 3 240	9 121 480 ± 635 237	985 201	708 503
thymus 4	156	F	thymus	1 848 851	1 345 927	3 812 418 ± 7 380	3 606 280 ± 5 422	6 826 960 ± 368 256	1 166 396	885 107
blood 1	7	M	blood	154 682	130 307	540 969 ± 4 198	540 969 ± 4 123	827 694 ± 56 037	121 367	108 361
blood 2	52	M	blood	123 523	103 142	430 223 ± 3 766	430 223 ± 3 700	681 737 ± 35 608	94 556	76 513
blood 3	107	M	blood	245 126	170 333	522 505 ± 3 003	522 505 ± 2 923	885 943 ± 47 301	114 452	5 901
blood 4	156	F	blood	199 326	157 728	56 686 ± 3 696	566 868 ± 3 615	1 270 020 ± 90 170	137 613	62 127

TCRB										
Sample id	Age (days)	Sex	Tissue	Observed counts	Observed diversity	Chao1 (mean±std)	Extrapolated ChaoE (mean±std)	Efron-Thisted (mean±std)	Shannon's index (mean)	Inversed Simpson's index (mean)

thymus A	243	M	thymus	1 647 656	1 245 029	3 463 693 ± 6 808	1,360,137 ± 899	5 568 945 ± 281 571	1 115 157	949 669
thymus B	244	M	thymus	1 783 878	1 526 694	7 198 338 ± 17 565	1,576,012 ± 1 097	11 613 883 ± 828 672	1 426 418	1 275 231
thymus C	225	F	thymus	1 850 299	1 551 603	6 175 838 ± 13 550	1 551 603 ± 1 077	11 065 398 ± 661 911	1 446 717	1 302 914
thymus D	126	M	thymus	1 726 796	1 449 881	6 208 706 ± 14 737	1 537 939 ± 1 056	9 910 366 ± 569 512	1 343 436	1 185 806
thymus 1	7	M	thymus	237 063	222 925	2 056 755 ± 18 593	1 215 349 ± 4 192	2 736 169 ± 206 873	217 609	209 474
thymus 2	52	M	thymus	182 356	172 746	1 737 599 ± 18 637	1 136 517 ± 5 332	2 180 279 ± 113 043	169 209	163 907
thymus 3	107	M	thymus	142 903	137 920	2 039 501 ± 29 774	1 215 583 ± 6 936	1 782 350 ± 140 139	136 060	133 205
thymus 4	156	F	thymus	128 228	121 483	1 269 967 ± 16 587	971 553 ± 6 988	1 569 435 ± 90 703	118 934	115 045
blood 1	7	M	blood	82 418	77 281	713 105 ± 10 949	658 522 ± 7 882	865 983 ± 57 876	75 069	70 167
blood 2	52	M	blood	73 945	69 404	704 739 ± 12 003	651 718 ± 8 696	796 697 ± 63 815	67 046	59 899
blood 3	107	M	blood	134 110	103 551	1 029 542 ± 14 292	787 853 ± 6 017	1 089 644 ± 87 860	47 612	1 278
blood 4	156	F	blood	88 901	81 935	790 042 ± 12 109	707 833 ± 7 921	898 294 ± 62 024	77 810	64 418

**Table 3:** Resampled diversity estimates.

TCRAD									
Sample id	Age (days)	Sex	Tissue	Observed counts	Observed diversity	Resampled Chao1 (mean±std)	Resampled Efron-Thisted (mean±std)	Resampled Shannon's index (mean)	Resampled inversed Simpson's index (mean)
thymus A	243	M	thymus	11 838 086	6 763 870	3 439 802 ± 48 656	1 928 183 ± 109 031	119 114	116 022
thymus B	244	M	thymus	12 849 473	7 419 245	3 717 184 ± 117 571	1 934 212 ± 165 908	119 461	116 728
thymus C	225	F	thymus	8 359 283	5 259 057	3 519 847 ± 71 955	2 023 854 ± 156 215	119 278	116 379
thymus D	126	M	thymus	11 063 464	6 610 182	3 799 437 ± 74 891	2 113 509 ± 195 602	119 460	116 538
thymus 1	7	M	thymus	3 179 774	1 984 292	2 131 831 ± 30 009	1 796 761 ± 112 417	117 634	114 042
thymus 2	52	M	thymus	1 747 487	1 198 677	1 703 786 ± 14 235	1 686 940 ± 56 182	116 726	113 381
thymus 3	107	M	thymus	2 158 043	1 230 436	1 302 763 ± 10 861	1 458 706 ± 13 181	114 778	110 665
thymus 4	156	F	thymus	1 848 851	1 345 927	2 227 657 ± 27 749	1 956 797 ± 46 144	118 117	115 234
blood 1	7	M	blood	154 682	130 307	515 756 ± 2 407	850 317 ± 49 399	101 160	92 134
blood 2	52	M	blood	123 523	103 142	430 223 ± 0	681 737 ± 0	94 556	76 513
blood 3	107	M	blood	245 126	170 333	421 347 ± 1 715	760 367 ± 58 240	71 403	5 814
blood 4	156	F	blood	199 326	157 728	506 377 ± 1 192	824 158 ± 38 655	95 097	51 910

TCRB									
Sample id	Age (days)	Sex	Tissue	Observed counts	Observed diversity	Resampled Chao1 (mean±std)	Resampled Efron-Thisted (mean±std)	Resampled Shannon's index (mean)	Resampled inversed Simpson's index (mean)

thymus A	243	M	thymus	1 647 656	1 245 029	2 402 723 ± 69 516	1 320 715 ± 8 691	72 359	71 675
thymus B	244	M	thymus	1 783 878	1 526 694	4 788 673 ± 151 470	1 437 422 ± 2 372	73 122	72 751
thymus C	225	F	thymus	1 850 299	1 551 603	4 582 751 ± 147 345	1 423 151 ± 2 423	73 119	72 760
thymus D	126	M	thymus	1 726 796	1 449 881	4 058 264 ± 145 518	1 413 346 ± 5 795	72 976	72 540
thymus 1	7	M	thymus	237 063	222 925	1 912 950 ± 45 644	1 275 516 ± 15 408	71 922	71 039
thymus 2	52	M	thymus	182 356	172 746	1 663 403 ± 52 735	1 238 325 ± 16 294	71 651	70 673
thymus 3	107	M	thymus	142 903	137 920	1 974 903 ± 20 177	1 263 684 ± 15 635	72 045	71 233
thymus 4	156	F	thymus	128 228	121 483	1 206 007 ± 23 326	1 135 274 ± 12 074	70 702	69 306
blood 1	7	M	blood	82 418	77 281	709 395 ± 4 431	816 448 ± 47 909	67 932	63 859
blood 2	52	M	blood	73 945	69 404	704 739 ± 0	796 697 ± 0	67 046	59 899
blood 3	107	M	blood	134 110	103 551	895 010 ± 8 921	798 491 ± 78 565	30 118	1 268
blood 4	156	F	blood	88 901	81 935	756 652 ± 5 086	741 337 ± 57 954	65 811	56 040

**Table 4:** Overlap measures.

## Ethics Statement

The study was approved by the Pediatric Ethical Committee of the Helsinki University Hospital (HUS/747/2019) and a written informed consent was obtained from the parents.

## CRediT Author Statement

NH and TPA conceptualised the study and wrote the original manuscript. NH and RV collected and prepared the samples. IK, DAY and JS implemented the software usage. IPM provided the study material. All authors reviewed and accepted the manuscript.

## Declaration of Competing Interest

The authors declare that they have no known competing financial interests or personal relationships which have, or could be perceived to have, influenced the work reported in this article.
